# Supplementary material for: Cells Dynamically Adapt Their Nuclear Volumes and Proliferation Rates During Single to Multicellular Transitions
Source: Adv Sci (Weinh). 2026 Mar 23;13(31):e24325. doi: 10.1002/advs.202524325 (PMC13588007; doi:10.1002/advs.202524325)
Supplement: Supplementary file 1 [file ADVS-13-e24325-s001.docx]

**Cells dynamically adapt their nuclear volumes and proliferation rates during single to multicellular transitions**

Vaibhav Mahajan^1^, Keshav Gajendra Babu^1^, Markus Mukenhirn^1,2^, Antje Garside^1^, Vinita Ajit^1^ Kini, Trishla Adhikari^1^, Timon Beck^1,3^, Byung Ho Lee^4^, Kyoohyun Kim^3^, Carsten Werner^5^, Raimund Schlüßler^1,6^, Alf Honigmann^1^, Sebastian Aland^7^, Anna Verena Taubenberger^1,5,*^

1. Dresden University of Technology, Biotechnology Center, Center for Molecular and Cell Biology, Tatzberg 47-49, 01307 Dresden, Germany
2. Department of Physiology, Development and Neuroscience, University of Cambridge, Downing Place, Cambridge CB2 3EL, United Kingdom
3. Max Plank Institute for the Science of Light, Staudtstraße 2, D-91058, Erlangen, Germany
4. Max Plank Institute of Molecular Cell Biology and Genetics, Pfotenhauerstraße 10, 01307 Dresden, Germany
5. Leibniz Institute of Polymer Research Dresden, Max Bergmann Center of Biomaterials Dresden, Hohe Str. 06, 01069, Dresden, Germany
6. CellSense Technologies, Ernst-Augustin-Straße 12, 12489 Berlin, Germany
7. Hochschule für Technik und Wirtschaft Dresden, Friedrich-List-Platz 1, 01069 Dresden, Germany

*Correspondence: Anna Taubenberger ([anna.taubenberger@tu-dresden.de](mailto:anna.taubenberger@tu-dresden.de))

**Supplementary data:**

*Figure S1: (A) Stiffness (Young’s modulus) of compliant MMP-cleavable, stiff cleavable and compliant non-cleavable PEG-heparin gels as measured by AFM (atomic force microscopy). Line in the box shows the median; box shows the 25th and 75th percentile; whiskers show the minimum and maximum values. n = 20 (compliant cleavable), 8 (stiff cleavable), 16 (compliant non-cleavable) gels. Each gel was prepared independently.*

*Figure S2: (A) Live confocal images of PANC1 H2B-mCherry in compliant MMP-cleavable PEG-heparin gels with CellMask Deep Red (membrane) at day 1, 4, 14. (B) Live confocal images of MCF10A expressing H2B-GFP in gels with CellMask Deep Red (membrane) at day 0, 1, 4, 7. (C) Cell volumes of HeLa FUCCI cells in gels at day 1 (absolute) and day 4 (average). n = 39 (day 1), 14 (day 4); 2-4 gels each; N = 1-2. Cyan bars represent medians. Mann-Whitney test was performed for statistical analysis. Scale bars – (A, B) 50 µm.*

*Figure S3: (A) Nuclear volumes of MCF-7 H2B-mCherry spheroids (day 14) in compliant MMP-cleavable PEG-heparin gels in relation to the distance of nuclei from the centre. n = 602; 3 spheroids. (B) Closest neighbour distance of the nuclei in MCF-7 H2B-mCherry spheroids in relation to their distance from the centre. n = 620; 3 spheroids. Linear regression was performed and goodness-of-fit was used to calculate R^2^.*

*Figure S4: (A) Cross-sectional area MCF-7 H2B-mCherry spheroids (day 14) in compliant MMP-cleavable, stiff cleavable and compliant non-cleavable PEG-heparin gels. Line in the box shows the median; box shows the 25th and 75th percentile; whiskers show the minimum and maximum values. n = 10 (compliant cleavable), 6 (stiff cleavable), 15 (compliant non-cleavable); 4 gels each; N = 2.*

*Figure S5: (A) Nuclear volume of MCF-7 H2B-mCherry in compliant non-cleavable PEG-heparin gels at day 1 and 4 under 10µm GsMTx-4 (Abcam - ab141871) (vehicle control - 41µL distilled water in 1mL media). Drug was added from the beginning. n = 13 (day 1 control), 16 (day 1 GsMTx-4), 69 (day 4 control), 46 (day 4 GsMTx-4); 2 gels each; N = 1. Cyan bars represent the median. Kruskal-Wallis test* *with multiple comparisons (Dunn’s) was performed for statistical analysis.*

*Figure S6: (A) Percentage of HeLa FUCCI cells in G1 and S/G2 phases in compliant non-cleavable PEG-heparin gels at day 1 and 4. n = 57 (day 1), 222 (day 4); 2-6 gels each; N = 1-3. (B) Percentage of MCF-7 FUCCI single cells released from compliant cleavable PEG-heparin gels and multicellular structures in G1 and S/G2 at day 1, 4, 14. n = 45 (day 1), 51 (day 4), 162 (day 14); 4-6 gels each; N = 2.*

*Figure S7: (A) Nuclear volumes of MCF-7 FUCCI free-floating aggregates (200, 300, 400 cells) and single cells taken from 2D culture. n = 63 (2D), 301 (200 cells), 238 (300 cells), 378 (400 cells); 4 aggregates each; N=1. (B) Percentage of MCF-7 FUCCI cells in G1 and S/G2 phases in free-floating aggregates composed of 200, 300, 400 cells. n = 67 (2D), 352 (200 cells), 336 (300 cells), 504 (400 cells); 4 aggregates each; N=1.*

*Figure S8: (A) Gene sets related to cell cycle obtained through GSEA with NOM p-val < 0.05 and FDR q-val < 0.25. (B) Heatmap of genes involved in cell cycle (obtained from leading edge analysis of cell cycle related gene sets) which are differentially expressed between MCF-7 at day 1 and day 4 in compliant MMP-cleavable PEG-heparin gels.*

*Figure S9: (A) Cell volumes of released single MCF-7 H2B-mCherry from compliant MMP-cleavable PEG-heparin gels at different culture timepoints. n = 33 (day 1), 34 (day 4), 45 (day 14); 4-6 gels each; N = 2. Cyan bars represent the median. Kruskal-Wallis test with multiple comparisons (Dunn’s) was performed for statistical analysis.*

*Figure S10: (A) Fold change of genes for cellular pumps/channels being differentially expressed between MCF-7 at day 1 and 4 in compliant MMP-cleavable PEG-heparin gels.*

*Figure S11: (A) Live confocal images of RPE1-FUCCI myc invading aggregates in compliant MMP-cleavable PEG-heparin gels. First, RPE1-FUCCI myc free-floating aggregates were formed by seeding 50 cells per well in a 96-well U-bottom plate after 2 days. These aggregates were embedded in compliant MMP-cleavable gels and imaged after 3 days. (B) Nuclear volumes of RPE1-FUCCI myc either present in the spheroid or invading out of the spheroid. n = 505 cells (spheroid), 389 cells (invading cells); 4 gels each; N = 2. Cyan bars represent medians. Mann-Whitney test was performed for statistical analysis. Scale bar – (A) 100 µm.*

*Figure S12: (A) Average nuclear volume in relation to spheroid area for MCF-7 H2B-mCherry spheroids (day 14) in compliant and stiff MMP-cleavable and compliant non-cleavable PEG-heparin gels. n = 10 (compliant cleavable), 6 (stiff cleavable), 15 (compliant non-cleavable); 4 gels each; N = 2. (B) Average (absolute for single cells) nuclear volume in relation to number of cells for MCF-7 H2B-mCherry (day 1, 4, 14) in compliant cleavable PEG-heparin gels. n = 53 (day 1), 89 (day 4), 8 (day 14); 4-10 gels each; N = 2-5. Linear regression was performed and goodness-of-fit was used to calculate R^2^.*

*Figure S13: (A) Nuclear volumes of MCF-7 H2B-mCherry cultured with 10 µm GsMTX-4 and 10 µm BAPTA from day 0 in compliant non-cleavable and MMP-cleavable (respectively) PEG-heparin gels at day 1 and 4. n=13-16 cells for day 1 and n=46-131 cells for day 4; 2 gels each; N = 1. Cells treated with 10 µm EIPA, 10 µm BAPTA-AM, 2 µm ionomycin and 5 mM EGTA did not survive till day 4. (B) Nuclear volumes of MCF-7 H2B-mCherry single cells released from compliant cleavable PEG-heparin gels at day 4 along with 10 µm EIPA, 25 µm DCPIB and 10 µm DFP00173. n=17-22 cells for released single cells and 66-90 cells for in-situ control; 2 gels each; N = 1. Cyan bars represent the median. Kruskal-Wallis test with multiple comparisons (Dunn’s) was performed for statistical analysis.*

*Figure S14: (A) Nuclear volume of MCF10A H2B-GFP spheroids (day 7) in compliant MMP-cleavable PEG-heparin gels in relation to the distance of nuclei from the centre. n = 139; 3 spheroids. Linear regression was performed and goodness-of-fit was used to calculate R^2^.*

*Table 1: Cell cycle phase (G1 and S/G2) lengths obtained from light sheet imaging of MCF-7 FUCCI in compliant MMP-cleavable PEG-heparin gels during days 1-4 and days 8-10 of culture time.*

| Day 2 to 4 | G1 length (hours) |  | S/G2 length (hours) | Day 8 to 10 | G1 length (hours) |  | S/G2 length (hours) |
| --- | --- | --- | --- | --- | --- | --- | --- |
| Entity 1 |  | **Entity 1** |  | **Entity 1** |  | **Entity 1** |  |
| Cell 1 | 9 | **Cell 1** | 18.5 | **Cell 1** | 23 | **Cell 1** | >17 |
| Cell 2 | 6.5 | **Cell 2** | 17.5 | **Cell 2** | 26.5 | **Cell 2** | >23.5 |
| Cell 3 | 7.5 | **Cell 3** | 22 | **Cell 3** | >42.5 | **Cell 3** | >22.5 |
| Cell 4 | 7.5 |  |  | **Cell 4** | >22 | **Cell 4** | 20.5 |
| Cell 5 | 9 | **Entity 2** |  |  |  |  |  |
| Cell 6 | >19 | **Cell 1** | 15.5 | **Entity 2** |  | **Entity 2** |  |
|  |  | **Cell 2** | 16 | **Cell 1** | >14 | **Cell 1** | 23 |
| Entity 2 |  | **Cell 3** | 19 | **Cell 2** | >17 | **Cell 2** | 27 |
| Cell 1 | >45.5 |  |  | **Cell 3** | >48 | **Cell 3** | 22 |
|  |  | **Entity 3** |  |  |  |  |  |
| Entity 3 |  | **Cell 1** | >13.5 |  |  |  |  |
| Cell 1 | 10 | **Cell 2** | 24 |  |  |  |  |
| Cell 2 | 10 |  |  |  |  |  |  |
| Cell 3 | 9 |  |  |  |  |  |  |
| Cell 4 | 7 |  |  |  |  |  |  |
|  |  |  |  |  |  |  |  |
| Entity 4 |  |  |  |  |  |  |  |
| Cell 1 | 12.5 |  |  |  |  |  |  |
| Cell 2 | 12.5 |  |  |  |  |  |  |
| Cell 3 | >32.5 |  |  |  |  |  |  |
